# Acknowledgement to reviewers of *Extracellular Vesicles and Circulating Nucleic Acids* in 2021

**DOI:** 10.20517/evcna.2022.01

**Published:** 2022-01-13

**Authors:** EVCNA Editorial Office

**Affiliations:** *EVCNA* Editorial Office, 245 E Main Street Ste 107, Alhambra, CA 91801, USA.

Rigorous peer-review is the corner-stone of high-quality academic publishing. The editors of *Extracellular Vesicles and Circulating Nucleic Acids* (*EVCNA*) would like to express their sincere gratitude to the following reviewers for their precious time and dedication, regardless of whether the papers were finally published in 2021 [Table 1].

**Table 1:** The reviewers in 2021

**Names**		
Oscar Wiklander	Aijun Wang	Wei Seong Toh
Weiliang Xia	Florent Mouliere	John Nolan
Vivian Hook	Sowmya Yelamanchili Venkata	Rafael Rosell
Mujib Ullah	David Meckes	Miroslaw Kornek
Jongki Cho	Wuzi Dong	Cristina Soriano-Ubeda
Shengjun Wang	Aurélie Ledreux	Suman Dutta
Yusuke Yoshioka	Lynne Bemis	Michael W. Pfaffl
David M. Lubman	Chulhee Choi	Victor Ugaz
Shannon Holliday	Xu Xun	Joris Robert Vermeesch
Mehmet Ali Ergun	Peter Bugert	Murray David M. Mitchell
Pietro Gentile	Shie Liang Edmond Hsieh	Johng Sik Rhim
Michael Graner	Janusz W. Rak	Moonchang Baek
Vito D’Agostino	Shilpa Buch	Guoping Li
Xianwen Wang	Feifan Zhou	Da Zhang
Isaac Kirubakaran Sundar	Zhaohui Huang	

## DECLARATIONS

### Copyright

©The Author 2022.

